# Supplementary material for: Deciphering spatial genomic heterogeneity at a single cell resolution in multiple myeloma
Source: Nat Commun. 2022 Feb 10;13:807. doi: 10.1038/s41467-022-28266-z (PMC8831582; doi:10.1038/s41467-022-28266-z)

Supplemental material

Merz, Merz, Wang, Wei et al.

Deciphering spatial genomic heterogeneity at a single cell resolution in multiple myeloma

Content:

Supplemental table 1: Summary of treatment in patients with longitudinal samples

Supplemental table 2: Summary of the differential expression analysis for each individual (see additional Excel file, p-values (Bonferroni corrected) derived from two-sided Wilcoxon Rank Sum test on y-axis)

Supplemental table 3: Summary of findings from whole-exome sequencing (WES) (see additional Excel file)

Supplemental figure 1: Similarity Weighted Nonnegative Embedding (SWNE) to identify gene modules across patients and locations

Supplemental figure 2: Prognostic significance of STMN1 and TUBA1B expression in the MMRF CoMMpass dataset

Supplemental figure 3: Gene set enrichment analysis comparing up- and downregulated genes from osteolytic lesion and bone marrow

Supplemental table 1: Summary of treatment in patients with longitudinal samples

Patient	Regimen	Lenalidomide	Bortezomib	Dexamethasone	Daratumumab	cycles
NDMM01	RVD	25 mg/d<br>PO<br>d1-14	1.3 mg/m^2<br>SC<br>d1, 4, 8, 11	20 mg/d<br>PO<br>d1-2, 4-5, 8-9, 11-12	-	4<br>21d<br>cycles
NDMM03	RVD				-	
NDMM06	Dara-RVD				16 mg/kg IV, d1,8,15	

RVD = Lenalidomide/ Bortezomib/ Dexamethasone
Dara = Daratumumab
PO = *per os*
SC = subcutaneous
IV = intravenous

Supplemental figure 1: Similarity Weighted Nonnegative Embedding (SWNE) to identify gene modules across patients and locations

(A) Similarity Weighted Nonnegative Embedding (SWNE, https://github.com/yanwu2014/swne) was used for nonnegative matrix factorization (NMF) to decompose the expression matrix into biologically relevant factors. Factors and cells are plotted onto a two dimensional space together with selected genes. A similarity matrix is used to smooth cell, factor and gene embeddings to ensure that cells that are close in the high-dimensional space are also close in the 2D visualization. As expected, malignant plasma cells from all analyzed patients clustered closely together. Among the identified factors, factor_5 had the strongest pulling effect on cells. (B) In accordance with our analyses using Seurat for plasma cells from individual patients, genes associated with proliferation (STMN1, TUBA1B, HMGB2 and H2AFZ) were the top genes contributing to factor_5 (B). Relative expression ranging from low (0, white) to high (1.00, red).

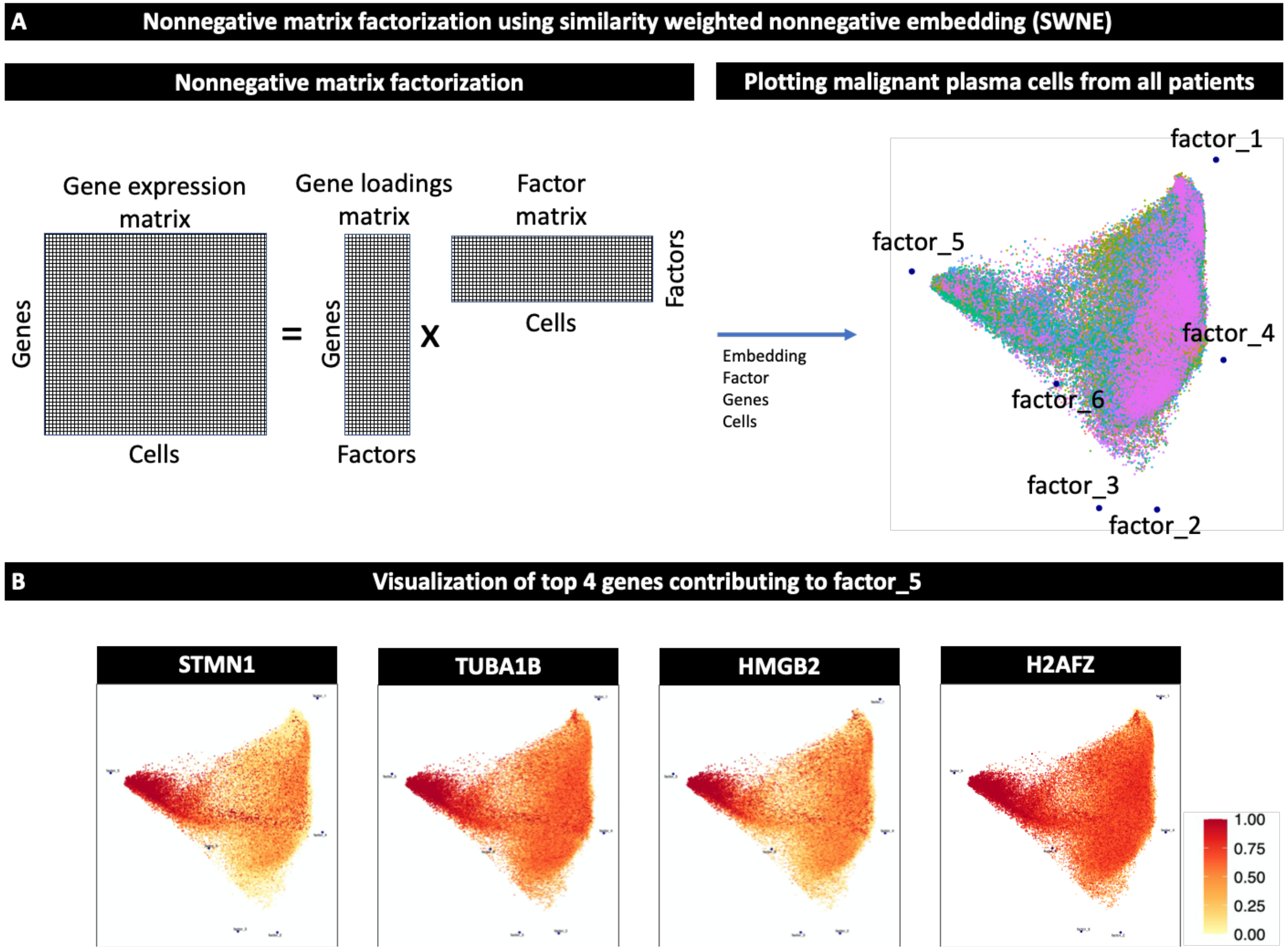

Supplemental figure 2: Prognostic significance of STMN1 and TUBA1B expression in the MMRF CoMMpass dataset

(A) Violin plots for TUBA1B and STMN1 expression for each individual patient. *XBP1* was included in the top row as positive control to show the extend of the entire PC population. Only a small subset of malignant PC co-expressed *STMN1* and *TUBA1B.* **(B)** Progression-free (PFS) for *STMN1* expression. **(C)** PFS for *TUBA1B* expression. Patients with lower expression in blue. Patients with higher expression in red. (**D**) The negative prognostic impact of STMN1 was reproduced in all patient groups, treated with a PI/IMID combination during induction or PI-based only therapy. P-values calculated with Kaplan-Meier analysis and two-sided log-rank tests. These data were generated as part of the Multiple Myeloma Research Foundation Personalized Medicine Initiatives using the Genospace Population Analytics platform (https://research.themmrf.org and www.themmrf.org)

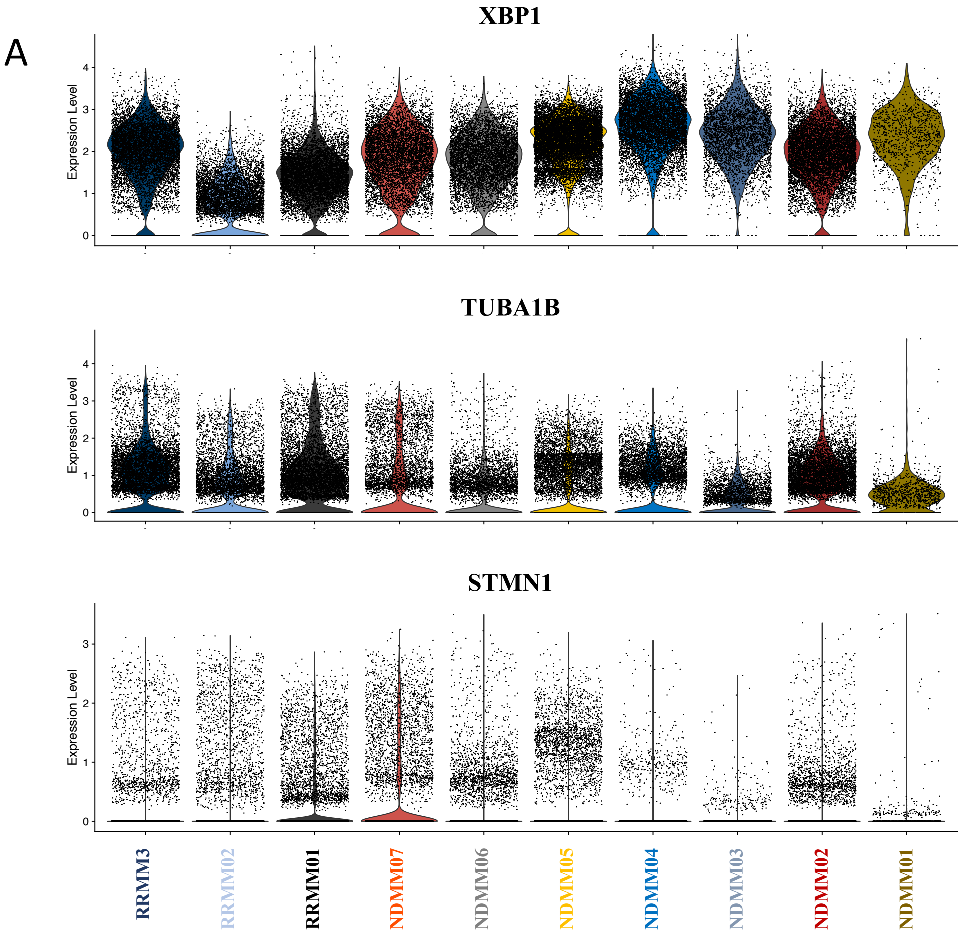

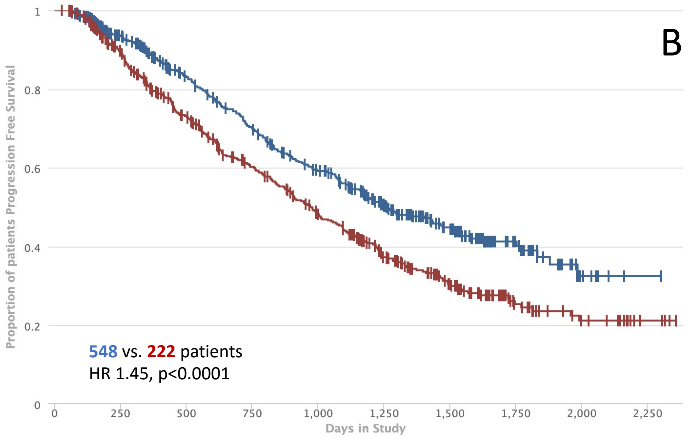

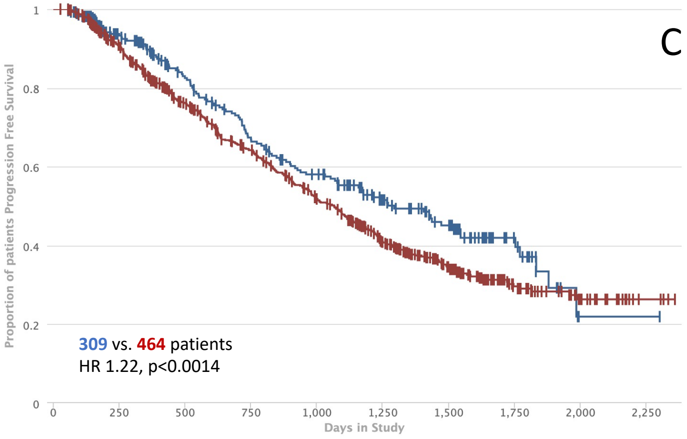

D

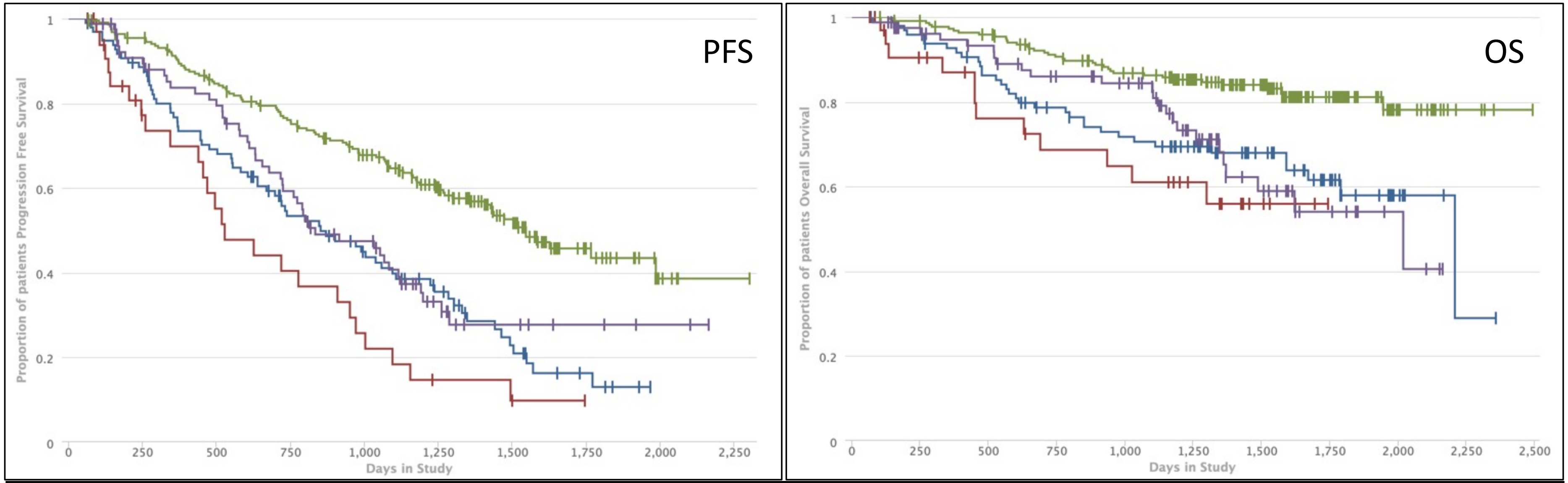

	Progression-free survival		Overall survival	
	Hazard ratio	p-value	Hazard ratio	P-value
STMN1 high and PI / IMiD combination	1.31	<0.0001	1.41	0.0003
STMN1 high and bortezomib-based	1.45	<0.0001	1.47	<0.0001
STMN1 low and PI / IMiD combination	0.64	<0.0001	0.63	<0.0001
STMN1 low and bortezomib-based	1.14	0.0327	1.31	0.0046

Supplemental figure 3: Gene set enrichment analysis comparing up- and downregulated genes from osteolytic lesion and bone marrow

Gene set enrichment analysis demonstrated that genes connected to regular B-cell function were significantly downregulated in OL. NES = normalized enrichment score. Padj = adjusted p-value estimation based on an adaptive multi-level split Monte-Carlo scheme

Pathway	NES	pval	padj
GO_DEFENSE_RESPONSE_TO_BACTERIUM	−3.19	1.0e−10	2.8e−07
GO_HUMORAL_IMMUNE_RESPONSE	−3.02	1.0e−10	2.8e−07
GO_RESPONSE_TO_BACTERIUM	−2.88	4.6e−10	8.5e−07
GO_ANTIMICROBIAL_HUMORAL_RESPONSE	−2.79	4.4e−09	6.0e−06
REACTOME_ANTIMICROBIAL_PEPTIDES	−2.56	6.9e−09	7.5e−06
GO_DEFENSE_RESPONSE_TO_OTHER_ORGANISM	−2.55	2.1e−08	1.9e−05
GO_INNATE_IMMUNE_RESPONSE	−2.52	6.0e−08	4.7e−05
GO_DEFENSE_RESPONSE	−2.38	1.4e−07	9.8e−05
GO_B_CELL_RECEPTOR_SIGNALING_PATHWAY	−2.63	3.0e−07	1.7e−04
REACTOME_METAL_SEQUESTRATION_BY_ANTIMICROBIAL_PROTEINS	−2.10	3.1e−07	1.7e−04

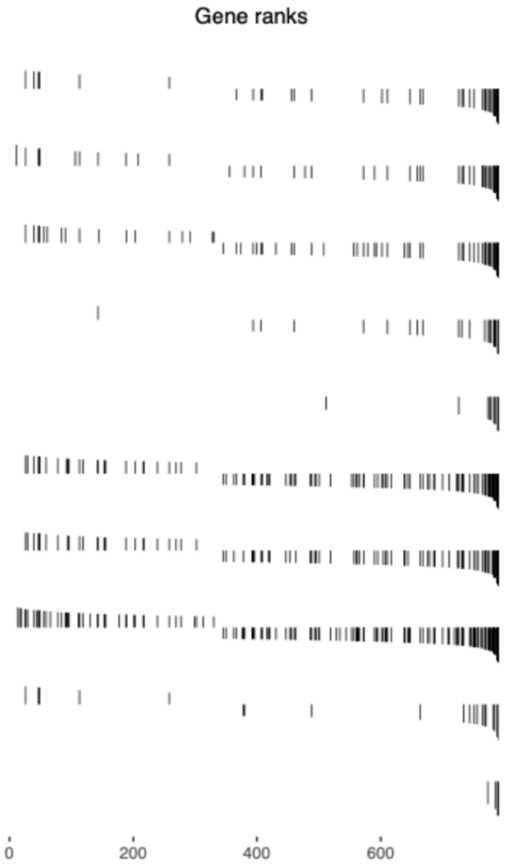

Supplement: Supplementary file 1 — Supplemental material [file 41467_2022_28266_MOESM1_ESM.pdf]
