# Supplementary material for: Deciphering spatial genomic heterogeneity at a single cell resolution in multiple myeloma
Source: Nat Commun. 2022 Feb 10;13:807. doi: 10.1038/s41467-022-28266-z (PMC8831582; doi:10.1038/s41467-022-28266-z)
Supplement: Supplementary file 5 — Reporting Summary [file 41467_2022_28266_MOESM5_ESM.pdf]

nature portfolio

Corresponding author(s): Maximilian Merz and Jens Hillengass

Last updated by author(s): Dec 13, 2021

Reporting Summary

Nature Portfolio wishes to improve the reproducibility of the work that we publish. This form provides structure for consistency and transparency in reporting. For further information on Nature Portfolio policies, see our Editorial Policies and the Editorial Policy Checklist.

Statistics

For all statistical analyses, confirm that the following items are present in the figure legend, table legend, main text, or Methods section.

n/a	Confirmed	
☐	☒	The exact sample size (*n*) for each experimental group/condition, given as a discrete number and unit of measurement
☐	☒	A statement on whether measurements were taken from distinct samples or whether the same sample was measured repeatedly
☐	☒	The statistical test(s) used AND whether they are one- or two-sided<br>*Only common tests should be described solely by name; describe more complex techniques in the Methods section.*
☒	☐	A description of all covariates tested
☒	☐	A description of any assumptions or corrections, such as tests of normality and adjustment for multiple comparisons
☐	☒	A full description of the statistical parameters including central tendency (e.g. means) or other basic estimates (e.g. regression coefficient) AND variation (e.g. standard deviation) or associated estimates of uncertainty (e.g. confidence intervals)
☐	☒	For null hypothesis testing, the test statistic (e.g. *F*, *t*, *r*) with confidence intervals, effect sizes, degrees of freedom and *P* value noted<br>*Give P values as exact values whenever suitable.*
☒	☐	For Bayesian analysis, information on the choice of priors and Markov chain Monte Carlo settings
☒	☐	For hierarchical and complex designs, identification of the appropriate level for tests and full reporting of outcomes
☐	☒	Estimates of effect sizes (e.g. Cohen's *d*, Pearson's *r*), indicating how they were calculated

Our web collection on statistics for biologists contains articles on many of the points above.

Software and code

Policy information about availability of computer code

Data collection	No specific code and software were used for data collection. The data depicted in this manuscript was generated by us.
Data analysis	CellRanger (v3.1.0), R(v4.0.0), Seurat (v3.2.2), SingleR (v1.8.0), Monocle3 (v1.0.0) , SWNE (latest version, so far no updates), LIGER (v0.5.0) , InferCNV (v1.3.3), EnhancedVolcano (v1.12.0) , FGSEA (v1.20.0) , ANNOVAR, SeqSQC (1.16.0), Piccard (2.22.), Bambino

For manuscripts utilizing custom algorithms or software that are central to the research but not yet described in published literature, software must be made available to editors and reviewers. We strongly encourage code deposition in a community repository (e.g. GitHub). See the Nature Portfolio guidelines for submitting code & software for further information.

Data

Policy information about availability of data

All manuscripts must include a data availability statement. This statement should provide the following information, where applicable:

- Accession codes, unique identifiers, or web links for publicly available datasets
- A description of any restrictions on data availability
- For clinical datasets or third party data, please ensure that the statement adheres to our policy

The raw scRNA-seq and WES data generated in this study have been deposited in the Sequence Read Archive (SRA) database under accession code PRJNA723584: [https://www.ncbi.nlm.nih.gov/bioproject/PRJNA723584/]

Field-specific reporting

Please select the one below that is the best fit for your research. If you are not sure, read the appropriate sections before making your selection.

☒ Life sciences ☐ Behavioural & social sciences ☐ Ecological, evolutionary & environmental sciences

For a reference copy of the document with all sections, see nature.com/documents/nr-reporting-summary-flat.pdf

Life sciences study design

All studies must disclose on these points even when the disclosure is negative.

Sample size	We performed an exploratory study and no sample size calculation was performed before analyzing 148,630 plasma cells from 24 different locations in 10 individuals with multiple myeloma (7 newly diagnosed, 3 relpased/refractory). The collected samples and numbers of samples were deemed sufficient since we mainly focused on intra-patient analyses and did not perform inter-group comparisons (e.g. NDMM vs RRMM, etc.).
Data exclusions	No samples were excluded. For single cell sequencing, we filtered out low quality or dying cells with more than 10% counts originating from mitochondrial genes. The cells detected with less than 500 or more than 7500 unique genes were also discarded to avoid empty droplets or multiplets. Furthermore, contaminating non-plasma cells were removed in silico after scRNA-seq for further analyses. Based on these steps, all consecutive samples collected in the trial could be used for further analyses.
Replication	scRNA-seq and WES experiments were performed once and not repeated since they met quality standards of the Roswell Park Comprehensive Cancer Center Genomics Shared Resources Core Facility.
Randomization	No randominzation was performed. Since we focused on intra-patient comparisons and did not compare treatments, specific subgroups or other interventions, randomizing patients was not relevant to our study.
Blinding	Since patients were not randomized to certain interventions, no blinding was performed.

Reporting for specific materials, systems and methods

We require information from authors about some types of materials, experimental systems and methods used in many studies. Here, indicate whether each material, system or method listed is relevant to your study. If you are not sure if a list item applies to your research, read the appropriate section before selecting a response.

Materials & experimental systems

n/a	Involved in the study	
☒	☐	Antibodies
☒	☐	Eukaryotic cell lines
☒	☐	Palaeontology and archaeology
☒	☐	Animals and other organisms
☐	☒	Human research participants
☐	☒	Clinical data
☒	☐	Dual use research of concern

Methods

n/a	Involved in the study	
☒	☐	ChIP-seq
☒	☐	Flow cytometry
☒	☐	MRI-based neuroimaging

Human research participants

Policy information about studies involving human research participants

Population characteristics	In April 2019 we initiated sample collection to analyze spatial and temporal evolution in newly diagnosed and relapsed MM. After written informed consent, patients underwent an imaging-guided biopsy of OL identified by PET/CT in addition to standard, diagnostic bone marrow aspirate from the iliac crest (BM). Biopsies were performed before the initiation of local or systemic therapy for newly diagnosed or relapsed patients. Eligible patients with a confirmed diagnosis of MM according to International Myeloma Working Group (IMWG) criteria were at least 18 years of age with an Eastern Cooperative Oncology Group (ECOG) performance status of 0-2 and no contraindications against general anesthesia. Key exclusion criterion was a history of other malignancy except if the patient had been symptom-free and without active therapy for at least 5 years. Patients were treated as standard of care and the procedure to obtain biopsies for this study did not affect their care plan. Seven patients had newly diagnosed and 3 patients relpased disease. Four patients were female, six patients were male. Median age at inclusion was 63 years and seven patients harbored IgG, two patients IgA and one patient light chain myeloma.
Recruitment	Patients were offered to participate in the trial if interventional readiologists identified at least one osteolytic lesion accesible for imaging-guided biopsy. No potential bias was intorduced by patient selection and we report results from the first 10 consecutive patients with paired samples. Patients did not receive financial compensation for participating in the study.

Ethics oversight	This study was approved by the Roswell Park Comprehensive Cancer Center (Roswell Park) Institutional Review Board and was conducted in accordance with the Declaration of Helsinki.

Note that full information on the approval of the study protocol must also be provided in the manuscript.

Clinical data

Policy information about clinical studies

All manuscripts should comply with the ICMJE guidelines for publication of clinical research and a completed CONSORT checklist must be included with all submissions.

Clinical trial registration	The study is registered at Roswell Park (I-66418).
Study protocol	The full study protocol can be requested from the corresponding authors.
Data collection	The study started in April 2019 and is ongoing. Data and sample collection are carried out by the authors.
Outcomes	Primary objective of the study is the investigation of spatial heterogeneity in multiple myeloma. Heterogeneity of the plasma cell compartment is assessed with scRNA-seq and WES as well as multi-color flow and histology.
